# Histological study of the annular ligament in the rabbitfish eye (*Siganus sp.*)

**Published:** 2012

**Authors:** Marziye Asli, Forooghsadat Mansoori, Amir Sattari

**Affiliations:** 1 *Graduate Student of Veterinary Medicine, Faculty of Veterinary Medicine, Shahid Bahonar University of Kerman, Kerman, Iran; *; 2 * Department of Basic Sciences, Faculty of Veterinary Medicine, Shahid Bahonar University of Kerman, Kerman, Iran; *; 3 *Department of Food Hygiene and Public Health, Faculty of Veterinary Medicine, Shahid Bahonar University of Kerman, Kerman, Iran.*

**Keywords:** *Siganus javus*, Annular ligament, Histology

## Abstract

Rabbitfish is economically valuable teleost species which lives in shallow coastal waters. Two species of rabbit fish have been recognized in southern sea of Iran (Persian gulf) as namely *Siganus sutor *and* Siganus javus.* In the current study, in order to investigate the histology of the annular ligament of the *S. javus’* eye, the prepared sections of the eyes of twelve healthy specimens were studied under light microscope. The results revealed that annular ligament is a crescent shape structure which is situated between the scleral stroma anteriorly and the iris posteriorly. It contains a vascularized, amorphous and granular matrix with fibers of dense connective tissue; high glycogen content and melanin pigments.

## Introduction

Annular ligament resembles a bracket shape structure situated between the cornea and the iris, fills much of the iridocorneal angle and runs circumferentially throughout the anterior chamber. Not strictly a ligament, it is named because provides some supportive structure to the anterior part.^1^^-^^2^ Although found in both cartilaginous and bony fishes but its presence is not universal like *corythoichthyes paxtoni *which does not possess a mesh-work of cellulo-fibrouse tissue as an annular ligament^3^^-^^5^ It supports a secretory function or controls the osmotic pressure in the aqueous humor,^5^ it may be refractive or metabolically active.^1^

In the current study, the structure of the annular ligament in *Siganus javus’* eye was investigated. This fish is economically valuable teleost species which lives in shallow coastal waters with aquatic plants. Two species of rabbitfish which has been recognized in Persian gulf are *S. sutor* and *S. javus.* Morphological, macroscopic and microscopic information about the fish tissues can help develop cellular biology and provide pathologic evaluation of diseases and lesions. Histological studies often play an important role to get these kinds of information.

## Materials and Methods

Twelve male rabbitfish with approximate weight 365 ± 100 g, where chosen from marine fish propagation and growing center of Bushehr province (South of Iran). All of them were healthy and in good conditions, without any external deformities and lesions. The fish were decapitated. Their heads were immersed in 10% neutral buffered formalin solution and kept for 7 days then their eyes were enucleated. Routine histological techniques were done and a 6 μm thickness sections were prepared. Sections were stained with standard Hematoxylin and Eosin (H&E), Masson Trichrome (TRI) and Periodic Acid-Schiff (PAS). Histological study was done using light microscope and photographs were taken for detailed illustration of the results.

## Results

**Fig. 1:**
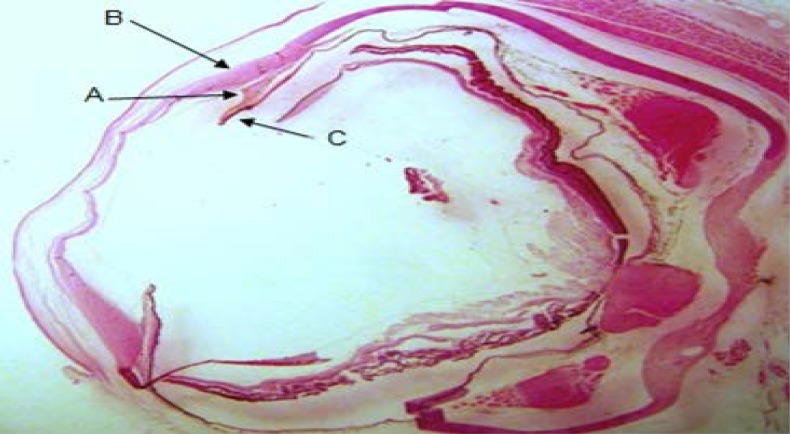
Photomicrograph of the *Siganus javus’* eye; **A.** Annular ligament ; **B.** Scleral stroma; **C.** Iris (H&E, 20×).

**Fig. 2:**
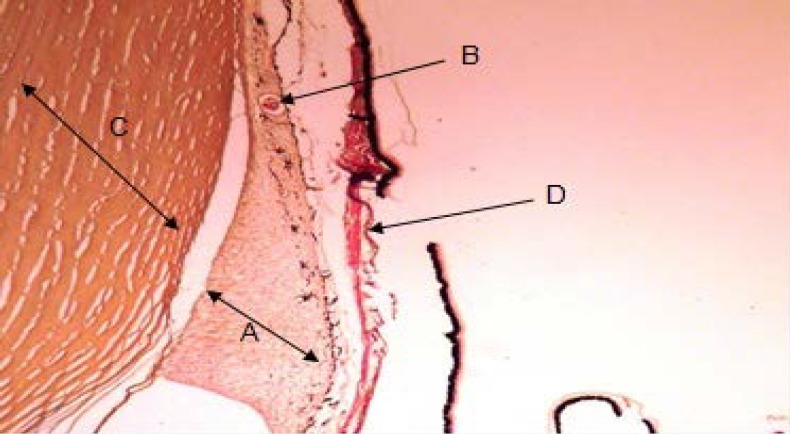
Annular ligament structure and position; **A.** Annular ligament with irregular fiber of connective tissue; **B.** Blood vessel; **C.** Fibers of scleral stroma; **D.** Iris (TRI, 100×).

**Fig. 3:**
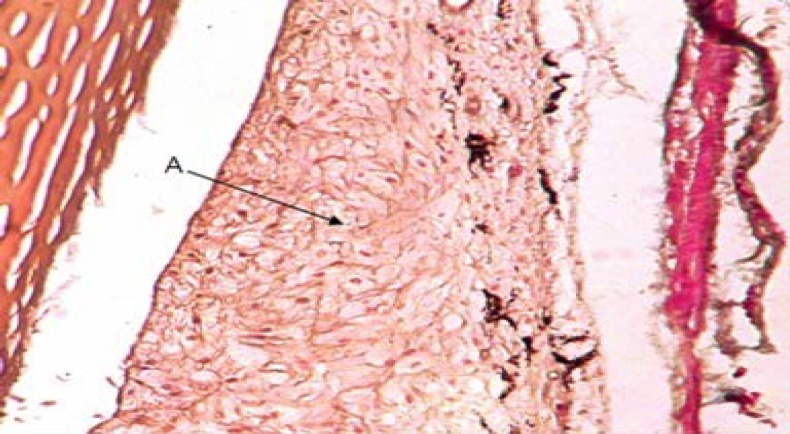
Annular ligament; **A.** Connective tissue (TRI, 400×).

**Fig. 4:**
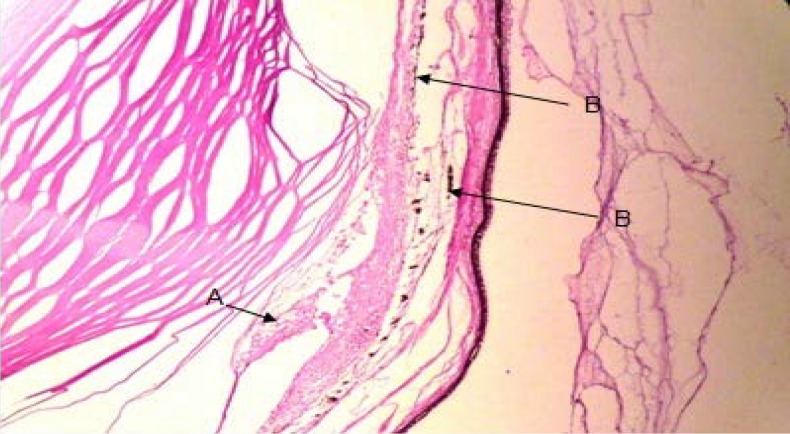
Photomicrograph of the annular ligament in *Siganus javus’* eye; **A.** Vacuolated cells between the connective tissue fibers; B. melanin pigments (H&E, 100×).

**Fig. 5:**
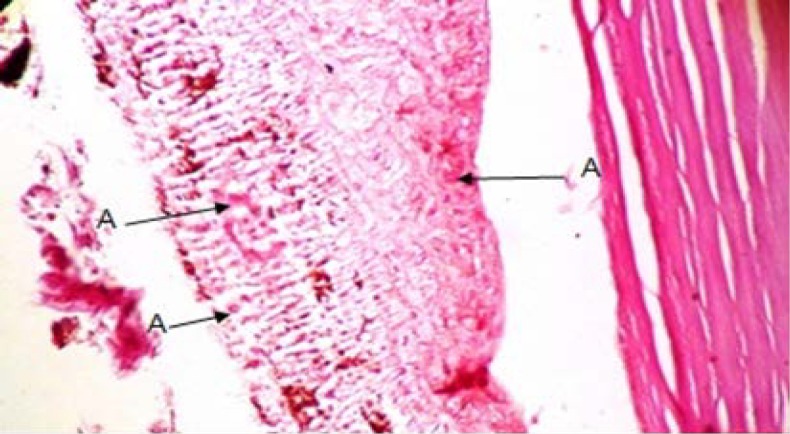
Annular ligament; **A.** Glycogen drops (PAS, 400×).

Annular ligament with a crescent shape was observed between the scleral stroma and iris in *Siganus javus’*eye. The anterior border was visible under the regular fibers of scleral stroma and there was not any layer as an endothelium over this structure. From the other aspect, the posterior border was situated over the iris (Fig. 1). It contained with an amorphous and granular matrix which was vascularized. It was filled with fibers of irregular dense connective tissue (Figs. 2 and 3). Staining with H&E and TRI revealed the presence of vacuolated cells between connective tissue fibers and PAS staining specified transparent spherical vesicles limited to a membrane as glycogen drops in these vacuoles (Figs. 3, 4 & 5). The observed melanin pigments at the posterior border confirmed the presence of melanophores that in some parts were sent out of the cells and scattered in matrix (Fig. 4).

## Discussion

Angelucci was the first one who described an accumulation of cells called the annular ligament.^6^ Duck-Elder claimed that it is a regular feature of the teleost cornea.^7^ Annular ligament embryologically is derived from and sometimes continues with the corneal endothelium.^4^^,5^ The surface of the annular ligament in zebra fish which is facing the anterior chamber is also lined by an endothelium but eye of *Siganus javus* does not have an endothelium layer and annular ligament is situated under the fibers of scleral stroma .This structure has a highly variable feature among the species. In the gold fish, *Carassius auratus*, it consisted of a collection of polyhedral cells filled with a granular material so it is called an annular meshwork.^4^ It is composed of a loose cellulofibrous tissue in the sturgeon and the garfish.^8^^,^^9^ In the mudskipper, it contains epithelioid cells which maybe vascularized, melanophores in the cod which is also observed in the annular ligament of the *Siganus javus’*eye.^4^ A rich glycogen content was seen in the *Siganus javus’* eye similar to that of salamander fish, *Lepidogalaxias salamandroides’ *eye suggesting metabolic role of this tissue which provides a source of energy for the cornea.^10^
